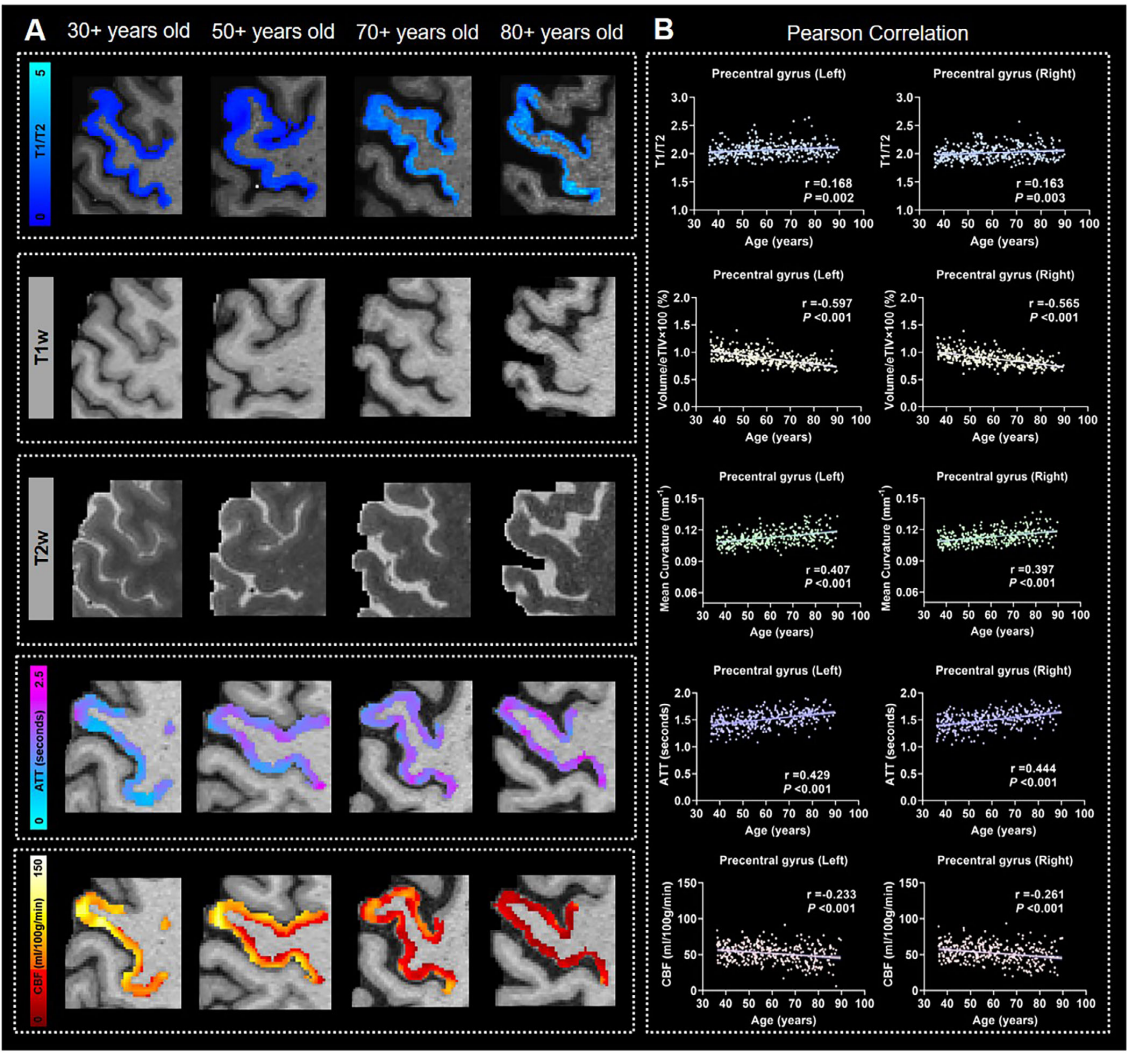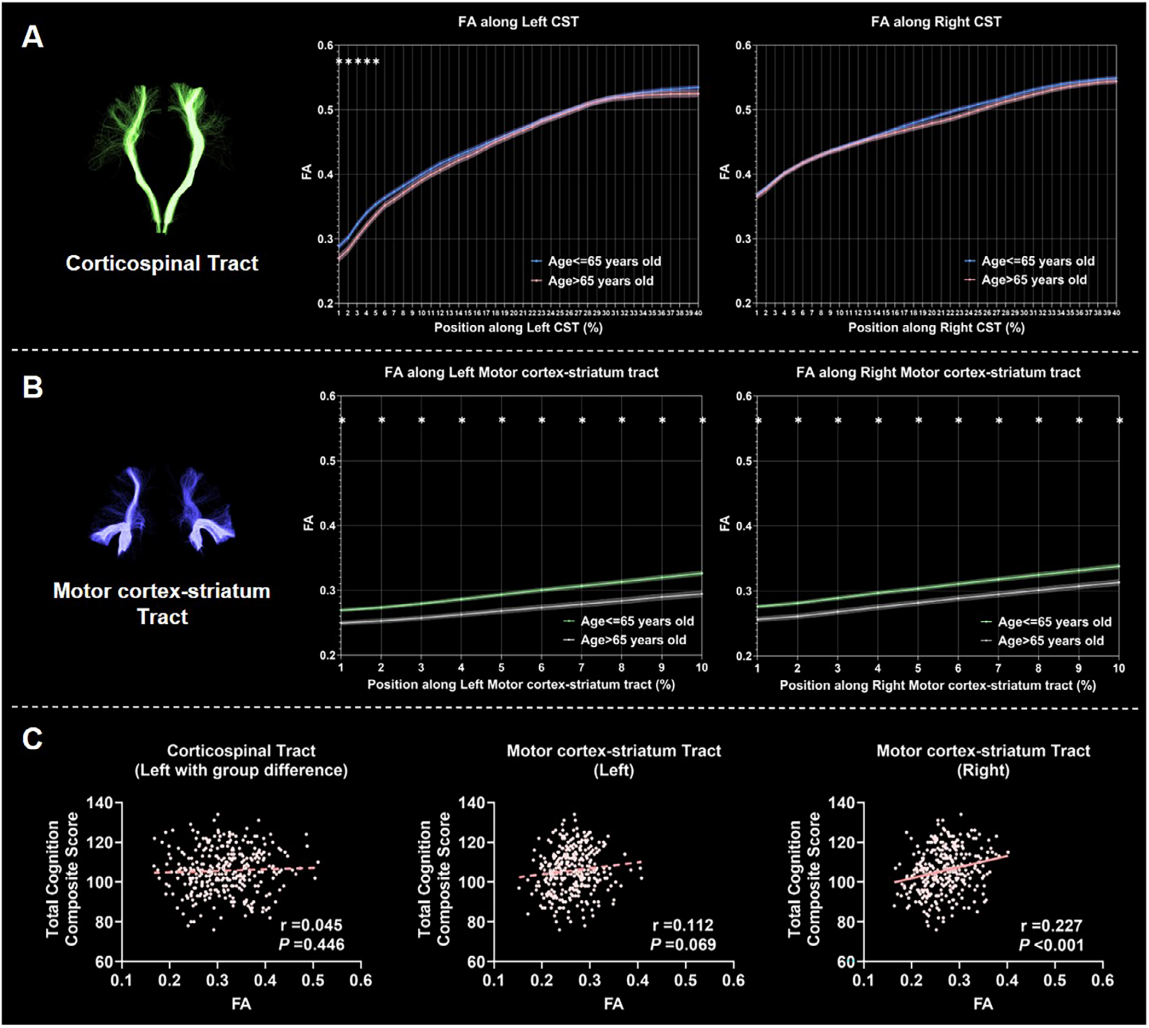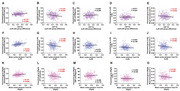# Age‐Related Degeneration of the Motor Cortex and White Matter Tracts: Insights from Structural and Perfusion MRI

**DOI:** 10.1002/alz70856_102308

**Published:** 2025-12-25

**Authors:** Jiaqi Wen, Zifei Liang, Chenyang Li, Huize Pang, Jiayi Li, Xiaojun Xu, Jiangyang Zhang, Yulin Ge

**Affiliations:** ^1^ New York University Grossman School of Medicine, New York, NY, USA; ^2^ The Second Affiliated Hospital, Zhejiang University School of Medicine, Hangzhou, Zhejiang, China; ^3^ NYU Grossman School of Medicine, New York, NY, USA

## Abstract

**Background:**

A decline in motor and cognitive function is a well‐recognized hallmark of aging in the elderly. In motor cortex (MC), pyramidal tract neurons, which project axons to the corticospinal tract (CST), also send collateral axons to the ipsilateral striatum, forming the MC‐striatum tract, a pathway potentially involved in cognitive functions. This study aims to comprehensively investigate the degeneration of the MC, CST, and MC‐striatum tract using advanced MRI techniques, providing new insights into the neural mechanisms underlying cognitive decline across a large‐scale adult lifespan population.

**Method:**

Data from 339 right‐handed subjects in the HCP‐Aging dataset were analyzed. MRI acquisition included T1‐MPRAGE, T2‐SPACE, pCASL, and diffusion sequences. Structural metrics (volume, curvature) were processed with FreeSurfer, and cerebral blood flow (CBF)/arterial transit time (ATT) maps were generated using hcpasl minimal processing pipeline. Tractography (CST, MC‐striatum tract) and along‐tract fractional anisotropy (FA) comparisons were conducted across age groups.

**Result:**

The 65–90‐year group exhibited lower cognitive composite score than the 36–65‐year group (*P* <0.001). The MC T1/T2 ratio and mean cortex curvature increased with age, while volume reduced (*P* <0.01, Figure 1). ATT was prolonged, and CBF was decreased in the MC with aging (*P* <0.001, Figure 1). The 65–90‐year group exhibited lower FA in left CST segments near the MC and the bilateral MC‐striatum tract compared to the 36–65‐year group (*P* <0.05, Figure 2). The mean FA of left CST segments near the MC and bilateral MC‐striatum tract positively correlated with MC volume and negatively correlated with the ATT and mean curvature (*P* <0.05, Figure 3). FA of right MC‐striatum tract was positively associated with the cognitive composite score (*P* <0.001, Figure 2).

**Conclusion:**

Aging is characterized by reductions in MC volume, CBF, and white matter integrity, alongside increases in T1/T2 ratio, curvature, and ATT. The MC‐striatum tract demonstrates a link to cognitive decline in aging, with its degeneration associated with structural and functional impairments in the MC. These findings provide insights into early alterations potentially linked to age‐related dementia and aid in their differential diagnosis.